# How competition governs whether moderate or aggressive treatment minimizes antibiotic resistance

**DOI:** 10.7554/eLife.10559

**Published:** 2015-09-22

**Authors:** Caroline Colijn, Ted Cohen

**Affiliations:** 1 Department of Mathematics, Imperial College London, London, United Kingdom; 2 School of Public Health, Yale University, New Haven, United States; University of British Columbia, Canada

**Keywords:** drug resistance, antibiotic, modelling, competition, other

## Abstract

Understanding how our use of antimicrobial drugs shapes future levels of drug resistance is crucial. Recently, there has been debate over whether an aggressive (i.e., high dose) or more moderate (i.e., lower dose) treatment of individuals will most limit the emergence and spread of resistant bacteria. In this study, we demonstrate how one can understand and resolve these apparently contradictory conclusions. We show that a key determinant of which treatment strategy will perform best at the individual level is the extent of effective competition between resistant and sensitive pathogens within a host. We extend our analysis to the community level, exploring the spectrum between strict inter-strain competition and strain independence. From this perspective as well, we find that the magnitude of effective competition between resistant and sensitive strains determines whether an aggressive approach or moderate approach minimizes the burden of resistance in the population.

**DOI:** http://dx.doi.org/10.7554/eLife.10559.001

## eLife digest

Antibiotics are chemical compounds used to treat bacterial infections. The discovery of antibiotics, starting with penicillin in 1929, revolutionized medicine, making it possible to cure or prevent life-threatening infections such as tetanus and pneumonia. However, many bacteria have become resistant to one or more antibiotics and so can no longer be killed by these drugs.

The emergence of antibiotic resistance reflects an evolutionary process that occurs during antibiotic treatment. While the antibiotic will kill most bacteria, some bacteria may naturally have a feature or genetic mutation that allows them to survive in the presence of the antibiotic. These bacteria then reproduce and pass on their resistant traits, eventually leading to the emergence of a new antibiotic-resistant strain of bacteria. Once a resistant strain exists it may be able to spread from one person to another.

There is conflicting evidence about how best to prevent antibiotic-resistant bacteria from evolving and spreading. The results of some experiments suggest that treating bacteria with large doses of antibiotics early in an infection is the most effective way to optimize treatment and minimize the risk of an antibiotic-resistant strain developing. However, other studies suggest that exposing bacteria to high levels of antibiotics more efficiently selects for resistance; in this case a more moderate approach should be used when treating bacterial infections.

Here, Colijn and Cohen present a mathematical model that suggests that the natural competition between the antibiotic-resistant and antibiotic-sensitive strains of bacteria influence which treatment strategy should be taken. Strains were modeled both within individual hosts and spreading in a community of individuals. In the models, aggressive antibiotic treatment is most effective (in that it minimizes antibiotic resistance) when the antibiotic-resistant strain either does not experience strong competition from the non-resistant strains of bacteria or is not fit enough to be a good competitor. However, a more moderate treatment is appropriate when the two strains are competing and the antibiotic-resistant strain is a fit competitor. Competition may mean that moderate treatment is best to avoid resistance at the community level, even in situations when aggressive treatment is likely best for individuals.

Two important future challenges are to better understand the diversity of strains in bacterial infections, and to develop tools to measure to what extent strains are effectively competing with each other.

**DOI:** http://dx.doi.org/10.7554/eLife.10559.002

## Introduction

The growing crisis of resistance to antimicrobial drugs has captured the attention of the global public health community as the harrowing reality of the loss of previously effective medicines combined with slow discovery of new agents threatens a post-antibiotic era of untreatable infectious diseases. Although the quality and completeness of surveillance is variable, current data are consistent with rising levels of resistance; this worrisome trend is not restricted to particular pathogens or specific geographic settings (WHO, 2014). While an accurate assessment of the current health and economic losses attributable to antibiotic resistance is elusive, the estimated numbers, ranging up to 2 million serious infections, 23,000 deaths, and 35 billion dollars in the United States alone, are staggering (CDC, 2013). Similar numbers of deaths have been attributed to antibiotic-resistant infections in Europe (O'Neill, 2014). Most recently, a projection of 10 million deaths and 100 trillion dollars in economic losses attributable to antimicrobial-resistant infections by 2050 has been circulated (O'Neill, 2014).

Given that antimicrobial treatment cures infections while simultaneously selecting for antimicrobial resistance, it is crucial to understand how alternative treatment strategies affect the probability of resistance. The conventional wisdom guiding the rapidity and dosing of drugs, often attributed to Paul Ehrlich, (Ehrlich, 1913) is that early and aggressive use of antimicrobial agents is most effective for optimizing cure and minimizing the risk of resistance (Levin et al., 1999). Recently, there has been some debate as to the universality of the claim that these aggressive approaches are optimal for minimizing the risk of resistance, with some researchers suggesting that more moderate approaches may perform better (Read et al., 2011) and others defending the standard approach (Ankomah and Levin, 2014).

A central rationale for an aggressive approach is that early high dose treatment will most rapidly reduce the size of the microbial population from which drug-resistant isolates appear and thus minimize the probability of the emergence of resistance during treatment (Bonhoeffer et al., 1997; Knudsen et al., 2003; Wiuff et al., 2003; Tam et al., 2005, 2007; D'Agata et al., 2007; Ankomah and Levin, 2014; Kim et al., 2014). In contrast, the rationale for a more moderate approach is that higher doses of antibiotics impose stronger selective pressure which drives a more rapid emergence of resistance (Read and Huijben, 2009; Read et al., 2011; Kouyos et al., 2014), and that rapid suppression of drug-susceptible isolates may allow for competitive release of existing drug-resistant isolates (de Roode et al., 2004; Wargo et al., 2007; Huijben et al., 2010; Pena-Miller et al., 2013; Pollitt et al., 2014). Recently, Kouyos et al. (2014) summarized the relevant, albeit limited, empirical evidence about dosing and risk of resistance, and described a ‘conceptual curve’ relating the strength of selection to the expected rate of resistance emergence, highlighting theoretical conditions under which aggressive and moderate approaches may be preferred.

How one formulates the question about optimal antimicrobial dosing strategies to minimize resistance will depend on one's perspective. For example, a clinician will likely be most concerned with identifying the dosing regimen that produces the best health outcome for the patient (i.e., highest probability of cure accounting for toxicities and the risk of resistance). A public health practitioner will likely seek to identify which treatment practices produce the greatest health gains while minimizing the long-term levels of resistance in the community. The recent debate over aggressive and moderate approaches has mainly been centered on identifying an optimal strategy for the treatment of individual hosts to minimize the probability of resistance. However, the emergence and subsequent transmission of resistance in the population may be of even greater concern. From a theoretical perspective, optimal dosing strategies for the prevention of resistance in individuals are not necessarily optimal for limiting resistance at the population level (Lipsitch and Samore, 2002; Mills et al., 2013).

Here, we provide a modeling framework that unifies the individual-level and population-level perspectives and provides additional insight into the debate about aggressive and moderate approaches for antimicrobial treatment. We demonstrate that the extent of effective competition between drug-susceptible and drug-resistant isolates is a key determinant of whether an aggressive approach is better (in terms of resistance prevention) than a moderate approach for hosts being treated for disease. Most importantly, we find that even within a model that allows for very strong competition, different realistic combinations of parameter values can support the aggressive or moderate approach as optimal. We illustrate how it is possible that models can support two such different conclusions by carefully considering the dominant interactions between the strains. We extend our analysis to the population level, exploring a spectrum of inter-strain interactions ranging from strict competition to independence. We find that the same framework explains why either aggressive or moderate treatment approaches can minimize resistance.

## Materials and methods

### Within-host model

We describe two populations of bacteria within a single host using a model based on Ankomah and Levin (2014). The model includes both wild-type (drug-sensitive; DS) bacteria and drug-resistant (DR) bacteria which arise by some presumably rare mechanism from the drug-sensitive type. This mechanism could be single-point mutation, acquisition of resistance genes through horizontal gene transfer, or another mechanism (Lipsitch and Samore, 2002; zur Wiesch et al., 2011). While we do not explictly model these differences, we note that the mechanisms of resistance and their probabilities affect the relative importance of de novo resistance compared to pre-existing resistance circulating in a population (Lipsitch and Samore, 2002). In our model, each strain initiates an immune response which follows density-dependent kinetics. Bacteria grow in a resource-dependent manner and have a death rate which increases under higher antibiotic concentrations. Antibiotics enter the system and degrade at a constant rate. The DR strain, by definition, has a higher minimum inhibitory concentration (MIC) than the DS strain and is assumed to have a slower growth rate reflecting fitness costs associated with mutation or acquisition of resistance genes. The phrase ‘minimum inhibitory concentration’ parallels the language in Ankomah and Levin (2014); the MIC is the value of antibiotic *A* at which the growth rate with the drug is half its baseline value (when *A* = 0). The strain interactions in the model are complex: strains compete for resources, and each strain can suppress the other by triggering a host immune response. Thus, we expect the strains to be under fairly strong competition. However, the DS strain also benefits the DR strain as DR is generated from the DS population through acquired resistance.

The equations are:

(1) $$\begin{array}{l} \frac{dB_{s}}{dt}=G_{s}B_{s}-k_{p}PB_{s}-k_{i}IB_{s}-\mu B_{s}\\ \frac{dB_{r}}{dt}=G_{r}B_{r}-k_{p}PB_{r}-k_{i}IB_{r}+\mu B_{s}\\ \frac{dI}{dt}=\alpha I\frac{B_{s}+B_{r}}{B_{s}+B_{r}+\sigma_{i}}-u_{i}I\\ \frac{dP}{dt}=\eta \left(P_{\text{max}}-P\right)\frac{B_{s}+B_{r}}{B_{s}+B_{r}+\sigma_{p}}-\gamma P\\ \frac{dR}{dt}=w\left(C_{R}-R\right)-e\left(G_{s}B_{s}+G_{r}B_{r}\right)\\ \frac{dA}{dt}=A_{\text{in}}-\left(d+w\right)A. \end{array}$$

The variables are the densities of DS and DR bacteria (*B*_*s*_, *B*_*r*_ in cells/ml) and the adaptive and innate immune cells (*I*, *P* in cells/ml), and the concentration of the resource *R* (*μ*mg/ml) and the antibiotic *A* (*μ*mg/ml). Bacterial growth is resource dependent (Ankomah and Levin, 2014) with growth rate $\lambda_{s}={\text{Λ}}_{s}\frac{R}{k+R}$ and similarly for *λ*_*r*_, where Λ_*s*_ and Λ_*r*_ are the maximum growth rates of the two strains when the resource is not limiting. The net growth rate *G*_*s*_ is *G*_*s*_ = *λ*_*s*_ − (*u*_*s*_ + *δ*_*s*_(*A*)) and *G*_*r*_ = *λ*_*r*_ − (*u*_*r*_ + *δ*_*r*_(*A*)), capturing death and the bactericidal effect of the antibiotic. We adopt the assumption that recruitment of activated effector cells of the innate system (*P*) is dependent on the density of the pathogen population (*B*_*s*_ + *B*_*r*_) and *σ*_*p*_ > 0 is a saturation constant. Similarly, we assume that the expansion of specific adaptive immune response *I* is dependent upon the density of the pathogen population, the maximal expansion rate *α* and *σ*_*i*_, the pathogen density at which the increase in the adaptive immune response is half maximal. The resource is replenished at rate *wC*_*R*_ and depleted at rate *wR*. In our baseline results we assume, as in previous work (Ankomah and Levin, 2014), that it is the net growth *G*_*s*_ and *G*_*r*_ that determine the extent of the depletion of resources. This means that if the net growth is negative, lysis of cells can replenish the resource. We assess the sensitivity of our findings to this assumption in Appendix 1 and find that the model's inter-strain dynamics and their dependence on the parameters are unaltered when the lysis effect is removed (in which case the resource equation in (1) reads *dR*/*dt* = *w*(*C*_*R*_ − *R*) − *e*(Λ_*s*_*B*_*s*_ + Λ_*r*_*B*_*r*_)). To incorporate the possibility of stochastic die-off of the DR population when its density is very low, the growth rate is 0 when the density is less than 30 cells/ml. Not allowing for stochastic die-off might allow resistant strains to enjoy an unrealistic advantage in the model due to its deterministic nature. Here, 30 cells/ml is approximately 10^−8^ times the maximum bacterial load, though this varies with the parameter choice. Antibiotic concentration *A* speeds the death of bacteria according to a saturating mechanism $\delta_{s}\left(A\right)=\lambda_{s}\frac{A/M_{s}}{A/M_{s}+1}$, and similarly for *R*, where *M*_*s*_ and *M*_*r*_ are the minimum inhibiting concentrations of antibiotic for the DS and DR strains, respectively. *A* is introduced through dosage *A*_*in*_ and is removed at rate *d* + *w* (Ankomah and Levin, 2014).

To explore these complex interactions, we drew 60,000 sets of parameters from ranges containing the values used previously (Ankomah and Levin, 2014) (see Table 1), spanning a range of strengths of the immune system (*k*_*i*_, *k*_*p*_, *η*, *α*), relative overall fitness of the DR strain (Λ_*r*_, *M*_*r*_), pre-existing DR bacilli, the growth rate of the DS strain (Λ_*s*_), and the mutation parameter *μ*. Each parameter was chosen uniformly at random from the ranges given in Table 1. All were held fixed, and the model was simulated under multiple dosages. This gave us an understanding of how treatment affected resistance at each set of parameters. We captured the relationship between increasing *A*_in_ and the maximum and total (integrated over time in the simulations) DR at each randomly drawn set of parameters.

**Table 1.** Parameters and ranges **DOI:** http://dx.doi.org/10.7554/eLife.10559.009

Symbol	Description	Range	Unit
Within-host model			
Λ_*s*_	Max growth rate (DS)	0.4–0.8	hr^−1^
Λ_*r*_	Max growth rate (DR)	(0.6–1) × Λ_*s*_	hr^−1^
*k*	Hill coeffient in bacterial growth	0.5–50.5	μg/ml
*u*_*s*_, *u*_*r*_	Death rates DS, DR	0.2	hr^−1^
*m*_*S*_	MIC (DS)	1	μg/ml
*m*_*R*_	MIC (DR)	1–8	μg/ml
*k*_*p*_	Rate of innate immune clearance	10^−7^ *−* 10^−5^	hr^−1^
*k*_*i*_	Rate of adaptive immune clearance	10^−5^ − 10^−3^	hr^−1^
*μ*	Rate of DR mutation	5 × 10^−9^ − 5 × 10^−7^	#/division
*α*	Recruitment of adaptive immunity	0.002–0.02	hr^−1^
*σ*_*i*_, *σ*_*p*_	Hill parameter in *I*, *P* dynamics	1000, 10,000	cells/ml
*u*_*i*_	Loss rate of *I*	5 × 10^−5^ − 5 × 10^−4^	hr^−1^
*γ*	Loss rate of *P*	5 × 10^−4^ − 5 × 10^−3^	hr^−1^
*η*	Recruitment of innate immunity	10^−5^ − 9 × 10^−4^	hr^−1^
*w*	Washout rate	0.2	hr^−1^
*C*_*R*_	Resource reservoir concentration	300–700	μg/ml
*e*	Use of resource per unit growth	5 × 10^−7^	μg/cell
*A*_in_	Antibiotic treatment	0–2.5	μg/(ml × 24 hr)
*d*	Loss rate of antibiotic	0.1	hr^−1^
Between-host model			
*β*_*x*_	Transmission parameter (DS)	1–4	months^−1^
*β*_*y*_	Transmission parameter (DR)	1 − *β*_*x*_	months^−1^
*κ*	Partial immunity coefficient	1	none
*κ*_*t*_	Treatment protection from DS	1 − 0.3*T*	none
*c*	Similarity coefficient	0–1	none
*u*	DS clearance without treatment	$\frac{1}{3}\beta_{x}$ − $\frac{2}{3}\beta_{x}$	months^−1^
*u*_*y*_	DR clearance	*β*_*y*_/*R*_01_ − *β*_*y*_	months^−1^
*T*	Intensity of treatment	0–1	none
*r*	Release of DS through treatment	0–0.1	months^−1^
*u*_max_	Max clearance DS under treatment	1.05 *β*_*x*_	months^−1^

Ranges are indicated with a − separating lower and upper values. Where a single value is given the parameter was fixed. Within-host parameter ranges contain the values used in Ankomah and Levin (2014). The relative growth of the resistant and sensitive strains (without treatment) can be modified either through k or Λ. We have chosen to vary the Λ parameters as the effect on relative fitness is linear. In the between-host model the value of *u* was chosen such that the DS strain has a basic reproductive number in Bonhoeffer et al. (1997) and Ankomah and Levin (2014). Similarly, *u*_*y*_ was chosen so that *R*_02_ ranges from 1 to *R*_01_, to ensure that the DR strain has a smaller maximum growth rate than the DS strain.

We determined whether ‘aggressive’ or ‘moderate’ therapy was the best approach according to which one minimized the overall (maximum and total) levels of resistance. If treatment is negatively correlated with resistance, then more treatment results in less resistance and an aggressive approach is best. Conversely, if the correlation is positive, then treatment drives increases in resistance, and a moderate approach is best (from a resistance standpoint). Accordingly, parameter sets in which resistance levels were negatively (*S* < −0.7) or positively (*S* > 0.7) correlated with antibiotic dosage as determined by the Spearman correlation *S* were classed as ‘aggressive is best’ or ‘moderate is best’; other results were classed as neutral. We removed parameter sets in which treatment does not succeed to avoid unfair inclusion of those parameter sets in which the long-term selective pressure of unsuccessful treatment drives resistance. In the main analyses, we assume that the threshold for successful treatment is defined as causing a >80% reduction in the maximum DS population; in sensitivity analyses, we vary this threshold and provide results from the full set of simulations in which such a threshold is not imposed (see Appendix 1).

### Between-host model

**Figure 1.**
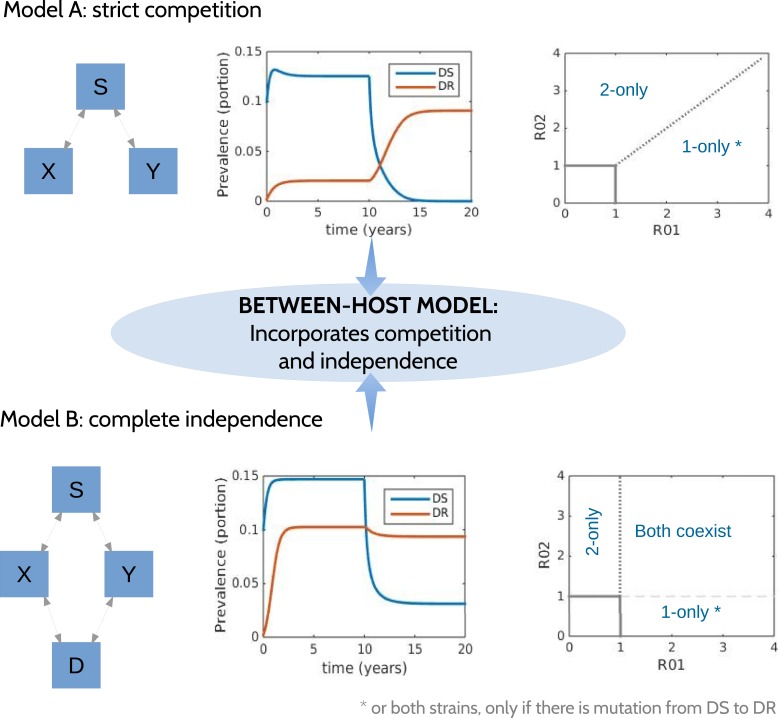
Models at the two ends of the competition independence spectrum (between hosts). In the competing model (top row), decreasing the DS strain (*X*) paves the way for an increase in resistance (*Y*) by removing the DR strain's competitor, despite the fact that decreasing DS also removes a source of resistance. The bifurcation analysis illustrates competitive exclusion: whichever strain has the higher *R*0 excludes the other. In both cases, some resistance is present when the sensitive strain is present, due to acquisition of resistance, for example, through mutation. Consequently, the ‘1-only’ region of the plot has some strain 2 at very low prevalence. In contrast, if the strains are not competing (bottom row), including not competing for hosts, hosts must be able to harbor both infections (duals, *D*). In this case, reducing DS reduces DR by reducing a source of resistance. The bifurcation plot where strains are independent illustrates that strain 1 is present if *R*_01_ > 1, strain 2 is present if *R*_02_ > 1 and both are present if they are both >1. Our between-host model incorporates competition and independence, parameterized by a ‘similarity coefficient’ that smoothly moves from one to the other (see Appendix 1 for details). **DOI:** http://dx.doi.org/10.7554/eLife.10559.010

To explore a wide range of inter-strain interactions at the population level, we developed a model with four host compartments: susceptible, infected with DS (*X*), infected with DR (*Y*) and dually infected (*D*). We envision a continuum of inter-strain interactions that in principle describe co-circulating pathogens. At one of the continuum, we posit that distinct pathogens may be entirely independent of each other, not interacting directly or indirectly (e.g., through immune modulation or resource competition). In this case, infection with one strain does not affect infection or recovery with the other strain. At the other end of the continuum, very similar strains of the same pathogen are likely to be competing for hosts. Figure 1 illustrates two models, one with strict competition and one with independence.

We have previously described ‘neutral null’ models (Lipsitch et al., 2009), in which biologically indistinguishable strains have sensible dynamics in models (i.e., outcomes do not depend on which strain a host has). Our model spans this continuum, which is parameterized by a ‘similarity coefficient’ *c*. When *c* = 1 the strains are highly similar and neutral in the sense of Lipsitch et al. (2009) if they are identical. When *c* = 0, the two strains act independently; infection with one does not affect the spread of the other. See Appendix 1 for more details and a proof of these statements.

The model equations are:

(2) $$\begin{array}{l} \frac{dX}{dt}=F_{x}S-\kappa F_{y}X+\frac{1}{2}\kappa_{t}cF_{x}D-u_{x}X+\left(1-c\right)u_{y}D-\left(\mu +Tr\right)X\\ \frac{dY}{dt}=F_{y}S-\kappa_{t}\kappa F_{x}Y+\frac{1}{2}\kappa_{t}cF_{y}D-u_{y}Y+\left(1-c\right)u_{x}D+\frac{1}{2}TrD+\left(\mu +Tr\right)X\\ \frac{dD}{dt}=\kappa F_{y}X+\kappa_{t}\kappa F_{x}Y-\frac{1}{2}\kappa_{t}c\left(F_{x}+F_{y}\right)D-\left(1-c\right)\left(u_{x}+u_{y}\right)D-\frac{1}{2}TrD-\frac{1}{2}c\left(u_{x}+u_{y}\right)D\\ u_{x}=u+\left(u_{\text{max}}-u\right)T,\;F_{x}=\beta_{x}X+\left(1-\frac{1}{2}c\right)D,\;F_{y}=\beta_{y}Y+\left(1-\frac{1}{2}c\right)D. \end{array}$$

In this model, hosts may become infected with both strains and enter the dually infected class (*D*); the chance of this may be reduced by cross-immunity, which we assume is symmetric between resistant and sensitive strains (*κ*). In models where dual infection cannot occur, there is an implicit assumption of very strong competition between strains. Dually infected individuals may also be again re-infected with a single strain (Lipsitch et al., 2009). Clearance terms (with recovery *u*_*x*_ and *u*_*y*_) are modulated with the similarity coefficient, *c*, to ensure that the model has independent interactions when *c* = 0 and neutral null dynamics when *c* = 1 (see Appendix 1). Transmission rates are *β*_*x*_ and *β*_*y*_, recovery rates are *u*_*x*_ and *u*_*y*_, and we assume that over the time frame of the simulation the population does not change; we scale it to 1 so that *S* = 1 − *X* − *Y* − *D*. The forces of infection *F*_*x*_ and *F*_*y*_ contain a contribution from both singly and dually infected hosts such that when the strains are different, dually infected hosts contribute as much as singly infected ones, and when they are very similar, each strain contributes half what a singly infected host would (Lipsitch et al., 2009). Treatment *T* ranges from 0 to 1 (where the DS strain is eliminated) and has several effects. Primarily, it cures the sensitive strain by reducing its duration of infection 1/*u*_*x*_. Individuals with a resistant strain (*Y*) who are given treatment are partially protected (*κ*_*t*_) from re-infection with the sensitive strain. Dually infected individuals given treatment have the drug-sensitive portion of their infection cured at an increased rate *u*_*x*_ due to treatment, but their resistant infection is not cured.

To capture the risk of releasing small sub-populations of resistant bacilli *within* such hosts, we include a parameter *r* which is a small rate at which resistance is uncovered by treatment. This parameter links the in-host and between-host models: in circumstances in which strong treatment drives increases in resistance, *r* would be high (approaching the treatment rate *u*_*x*_ of the sensitive strain).

We use a range of parameters such that the basic reproductive numbers (*β*/*u*) of the strains, *R*_01_ (DS) and *R*_02_ (DR), are greater than 1, with *R*_02_ < *R*_01_ (Table 1). We draw parameters randomly and increase the treatment *T*. We explore the relationship between the strength of treatment and the long-term and maximum level of resistance. We classify the resulting optimal strategy as aggressive if the Spearman correlation is less than −0.7 and moderate if it is larger than 0.7.

The between-host model admits three possible steady states (equilibria): a disease-free equilibrium (*X* = *Y* = *D* = 0 stable when *R*_01_ and *R*_02_ are both less than 1), a resistance-only steady state (*X* = *D* = 0, *Y* = 1 − *u*_*y*_/*β*_*y*_), and a steady state with *X*, *Y*, and *D* positive whose explicit form is not available. If there were no acquisition of resistance (*μ* = 0) and no ‘competitive release’ term (transition from *X* to *Y*), there would be an additional equilibrium with *Y* = *D* = 0 and *X* = 1 − *u*_*x*_/*β*_*x*_. We carried out an invasion analysis to determine the point at which the resistance-only equilibrium loses local stability as *β*_*x*_ increases. When the two strains are independent, *X* should be prevalent if and only if *R*_01_ ≥ 1. But if they are not, the values of the strain *Y* parameters (and its prevalence) affect the prevalence of *X*. We performed the invasion analysis as follows: we computed the Jacobian of model (2) and evaluated it at the resistance-only steady state for a given set of parameters including *β*_*y*_. We used matlab's nonlinear solver, fsolve, to determine the value of *β*_*x*_ at which the resistance-only steady state loses stability. We repeated this for a range of values of *β*_*y*_ to produce the colored lines in Figure 5C.

### Determining the role of parameters in defining whether treatment increases or decreases resistance

**Figure 2.**
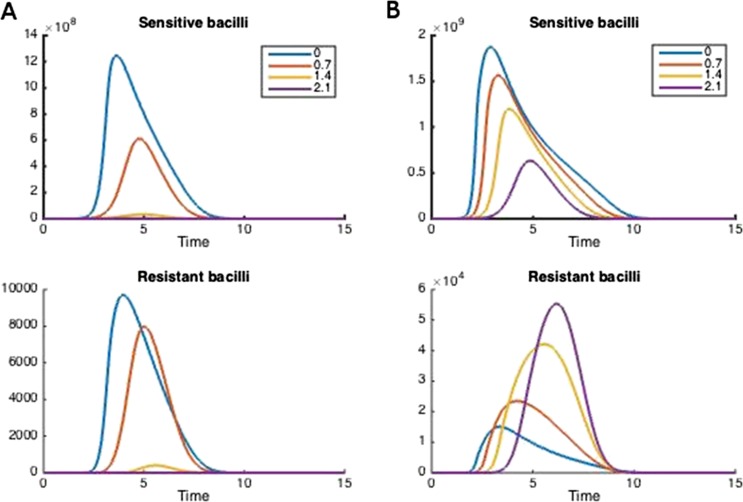
How treatment changes the trajectory of the in-host model. Parameters are (**A**) Λ_*s*_ = 0.5, Λ_*r*_ = 0.45, *m*_*R*_ = 2.8 and (**B**) Λ_*s*_ = 0.6, Λ_*r*_ = 0.53, *m*_*R*_ = 3.4. Other parameters are *k* = 48, *k*_*p*_ = 2.2*e*^−6^, *k*_*i*_ = 7.2*e*^−4^, *μ* = 4*e*^−7^, *α* = 0.018, *σ*_*i*_ = 1000, *σ*_*p*_ = 10000, *γ*_*p*_ = 0.001, and the remainder are as in Table 1. **DOI:** http://dx.doi.org/10.7554/eLife.10559.003

**Figure 3.**
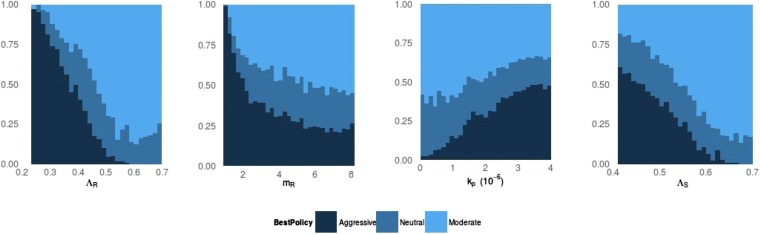
Frequency of best policies over key parameters. An aggressive policy (dark blue) is deemed best if the Spearman correlation *S* between treatment and resistance is *S* < −0.7, moderate (light blue) is deemed best if *S* > 0.7 and the classification is neutral (medium blue) otherwise. When the DR strain has a lower growth rate (LamR), an aggressive policy is more likely best because more of the DR strain's population arises through resistance acquisition from the DS population. In this case, reducing the DS strain also reduces DR. Conversely, when Λ_*R*_ (LamR) is high the DR strain is a more robust competitor and a moderate policy is more frequently best. Similarly, when the DR strain has a low MIC (mR), it is a less robust competitor. In this case, an aggressive policy is more frequently best than when mR is high (second panel). The third panel shows that when the immune system is strong (high *k*_*p*_), an aggressive policy is more frequently best, because again more of the DR population increases are driven by acquisition from DS, due to immune suppression of DR growth. A plot with *η* on the horizontal axis is very similar to this one. Finally, the right plot shows that when the DS growth rate (LamS) is low, an aggressive strategy is more often best to minimize resistance; this depends on the ability of therapy to prevent the emergence of resistance. **DOI:** http://dx.doi.org/10.7554/eLife.10559.004

**Figure 4.**
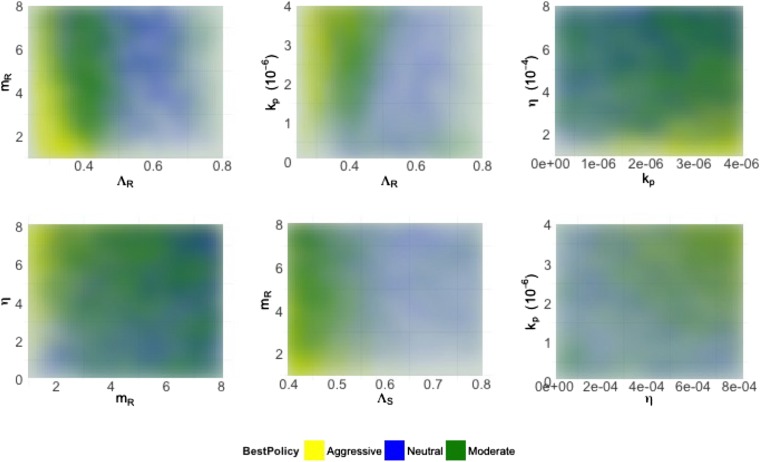
Heatmaps illustrating how best policies depend on key combinations of parameters. Color indicates the policy that minimizes resistance. Yellow: aggressive; green: neutral; blue: moderate. When the growth rate Λ_*R*_ (LamR) is high, a moderate policy is more frequently best, but a strong immune system (high *k*_*p*_) can compensate by reducing DR growth. When the DR strain is a strong competitor, a moderate policy is frequently best; this can be achieved by *either* a high Λ_*R*_ or a high DR MIC (mR) (top left). Either a high *k*_*p*_ or a high *η* can compensate (bottom right), reducing the growth potential of the DR strain and leading to either a neutral outcome or an aggressive policy being best. **DOI:** http://dx.doi.org/10.7554/eLife.10559.005

**Figure 5.**
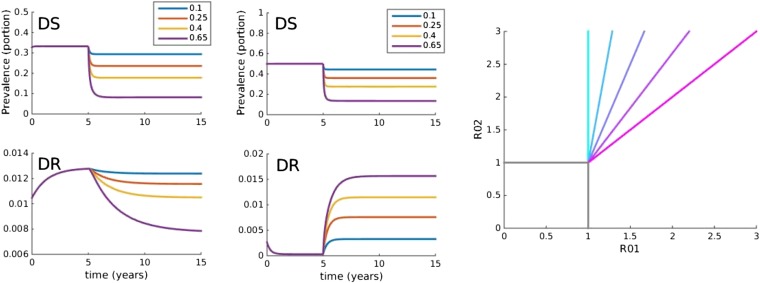
Trajectories of the between-host model under varying treatment. Treatment is introduced at 5 years. (Left) Parameters are *β*_*x*_ = 1.5, *β*_*y*_ = 1.04, *c* = 0.05, *r* = 0, *μ* = 0.001. (Middle) Parameters are *β*_*x*_ = 2, *β*_*y*_ = 1.1, *c* = 0.3, *r* = 0.05, *μ* = 0.0001. (Right) Invasion analysis (bifurcation) plot. The plot shows regions of stability of the disease-free equilibrium (both *R*_0_ values less than one), the DR-only equilibrium (top left region), and the equilibrium with both (primarily DS, with low-level DR due to acquisition). The diagonal lines show the boundary at which the DR-only equilibrium loses stability. Lines move to the right as the similarity coefficient increases from 0 (light blue vertical line) to 1 (pink). When it reaches 1, the line is *R*_01_ = *R*_02_. **DOI:** http://dx.doi.org/10.7554/eLife.10559.006

**Figure 6.**
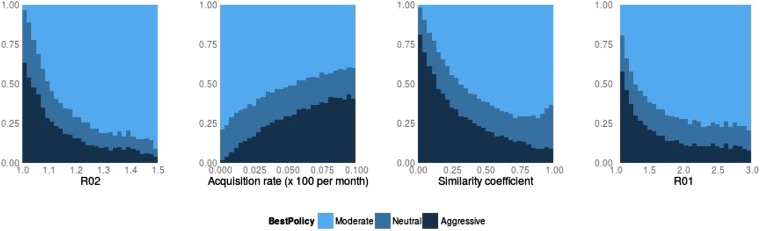
Best policy over parameters in the population-level model. Light blue corresponds to Spearman correlation greater than 0.7, dark corresponds to less than −0.7 and mid-range blue corresponds to all values in between. An aggressive policy is best when the DR strain is relatively unfit (low *R*_0_ value; left panel). When the acquisition rate is high, treatment-driven reductions in DS decrease the DR prevalence (second panel). When the two strains are more independent (low similarity coefficient), competition is reduced, so that reductions in the DS strain do not much benefit the DR strain, leading to the aggressive policy being preferred (third panel). When *R*_01_ is high, a moderate outcome results, because inter-strain competition is more effective. Vertical axes (‘count’) are the fraction of simulations in each category. **DOI:** http://dx.doi.org/10.7554/eLife.10559.007

**Figure 7.**
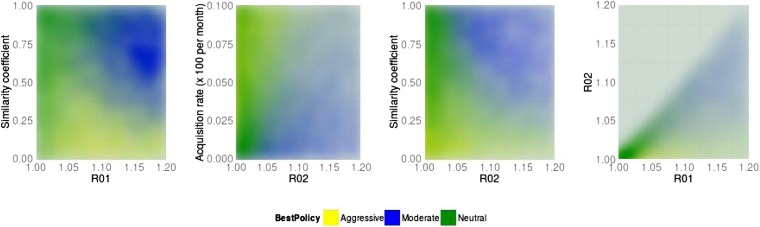
Best policy over parameter combinations in the population-level model. A moderate-is-best (blue) outcome occurs when *R*_01_ and the similarity coefficient are high, because the strains are competing (left panel). When the DR strain is relatively unfit (low *R*_02_), an aggressive policy is likely best (second and third panels); we restricted *R*_02_ to be lower for the DR strain than the DS one. A high rate of acquisition can counter-balance a low *R*_02_ (second panel). When the strains are more independent (low similarity coefficient), an aggressive policy is more often best even over a much wider range of *R*_02_ (third panel). **DOI:** http://dx.doi.org/10.7554/eLife.10559.008

We took several approaches to understand how the parameters of each model relate to whether aggressive or moderate treatment minimizes resistance. The most direct approach is simply to choose a set of parameters, vary the dosage, and examine how resistance changes (Figure 2). Naturally, the result depends strongly on the parameter choice. We also vary one parameter at a time, keeping others fixed, and examine the trajectories (Appendix figures 2, 3). The next approach is to examine, over all simulations simultaneously, how the outcome depends on each parameter by stratifying the outcomes (Figure 3). Using heatmaps or scatter plots, it is also possible to explore how *pairs* of parameters determine an outcome (Figure 4). We take the same approach in the between-host model, with Figure 5 showing demonstrative trajectories under varying treatment strength, Appendix figure 4 showing a sensitivity analysis varying one parameter at a time, and Figures 6, 7 showing the stratified dependence of the outcome on single and paired parameters while other parameters are allowed to vary.

However, we cannot expect any one or two parameters to entirely determine which approach minimizes resistance. We used discriminant analysis of principal components (DAPC, in the adegenet package in R [Jombart and Ahmed, 2011]) to systematically identify which parameters contribute to each outcome (Jombart et al., 2010). DAPC is related to principal component analysis (PCA) but instead of finding combinations of parameters to account for the variability in data (as PCA does), DAPC finds combinations of parameters that best account for variability *between groups*. Here, we used whether aggressive or moderate treatment minimizes resistance to define the groups (aggressive, neutral or moderate as above) and used DAPC to find combinations of parameters that separate these groups from each other.

## Results

The best policy for reducing the amount of resistance in both models can be either aggressive or moderate despite the fact that both models capture potentially strong competition and the acquisition of de novo resistance. The choice of best approach depends on the combined effect of the complex set of inter-strain interactions, and typically, one of these approaches is the clear winner (see Appendix figure 5).

### Within-host model

Figure 2 illustrates how treatment dosage may affect the level of resistance in the in-host model. Whether treatment increases or decreases resistance over the course of infection depends on many of the parameters in the model. We performed a sensitivity analysis to determine how the relationship between resistance and dosage changes with each parameter when the others are held fixed (Appendix figures 2, 3). We found that the relationship is relatively robust to variation in each parameter alone, but that small changes in several parameters can have a radical effect. In Appendix figure 2, increasing the dosage does not increase resistance and this result is fairly robust to variation in each parameter alone; Appendix figure 3 shows the contrasting robust scenario (increased dosage increases resistance). The difference is that the baseline parameters in Appendix figure 3 have somewhat higher growth Λ_*s*_ and Λ_*r*_ of both strains and the resistant strain has a higher MIC.

No single parameter determines whether aggressive or moderate treatment minimizes resistance. A relatively small change in several parameters can shift the model from one mode to the other. We separated parameter combinations in which aggressive vs moderate treatment minimizes resistance using a principled approach (DAPC; Appendix figure 6). We found that a linear combination of parameters (one ‘principle component’) captures almost all of the difference between the three groups (aggressive minimizes DR, moderate minimizes DR, or neutral, i.e., in between). The ‘loadings’ (e.g., coefficients) of the parameters in this linear combination correspond to the relative importance of the parameters in determining whether aggressive or moderate treatment minimizes resistance. The number of pre-existing resistant cells affects the outcome (coefficient 0.79). When pre-existing numbers are low (<10), parameters which lead to a ‘moderate’ policy to be best include higher growth rate of the DR and DS strains (Λ_*R*_, 1.4 and Λ_*S*_, 0.92), lower immune parameters *k*_*p*_ and *η* (−0.74; −0.66) and a higher MIC of the DR strain (mR; 0.34). Other parameters had loadings less than 0.1 and did not contribute much to the classification.

In other words, we find that an aggressive approach is preferred when the immune system is relatively strong (higher values of immune parameters *k*_*p*_, *η*), and when the DR strain has a relatively low growth rate (Λ_*R*_) and low MIC (*m*_*r*_). Conversely, if there is pre-existing resistance, the immune system is weaker, and/or the growth rate or MIC higher, a moderate approach minimizes resistance. Pre-existing populations of DR pathogens (i.e., resistance that appears prior to exposure to treatment) favor a moderate approach, since there is no possibility that a hard-and-fast approach will clear the infection before resistance can arise.

Figure 3 illustrates how the best policy relates to individual model parameters, and Appendix figures 7, 8 show the distributions of individual parameters where treatment increases or decreases resistance. No single parameter determines which policy is best; rather, the outcome depends on the combined effects of a set of complex interactions. This means that from a location in parameter space where aggressive therapy minimizes resistance, a relatively small change in several parameters (e.g., a slight decrease in *k*_*p*_, increase in *m*_*R*_ and increase in Λ_*r*_) can result in a moderate policy being best.

Figure 4 shows the best policies for key pairs of parameters whose values combine to influence whether an aggressive or moderate policy minimizes resistance. These figures reveal a few intuitive trade-offs: a higher DR growth rate (Λ_*R*_) generally leads to a moderate policy being best (light blue; positive correlation between treatment and DR), but this can be offset with a strong immune system keeping both strains in check (high *η* or high *k*_*p*_). A higher DR growth rate *or* a higher MIC (*m*_*R*_) make the DR strain a robust competitor and also consequently favors a moderate policy. Interestingly, while the mutation rate affects the overall numbers of DR bacteria (particularly in cases when they are not present initially), it does not have a strong effect on the relationship between treatment and total resistance.

**Appendix figure 1.**
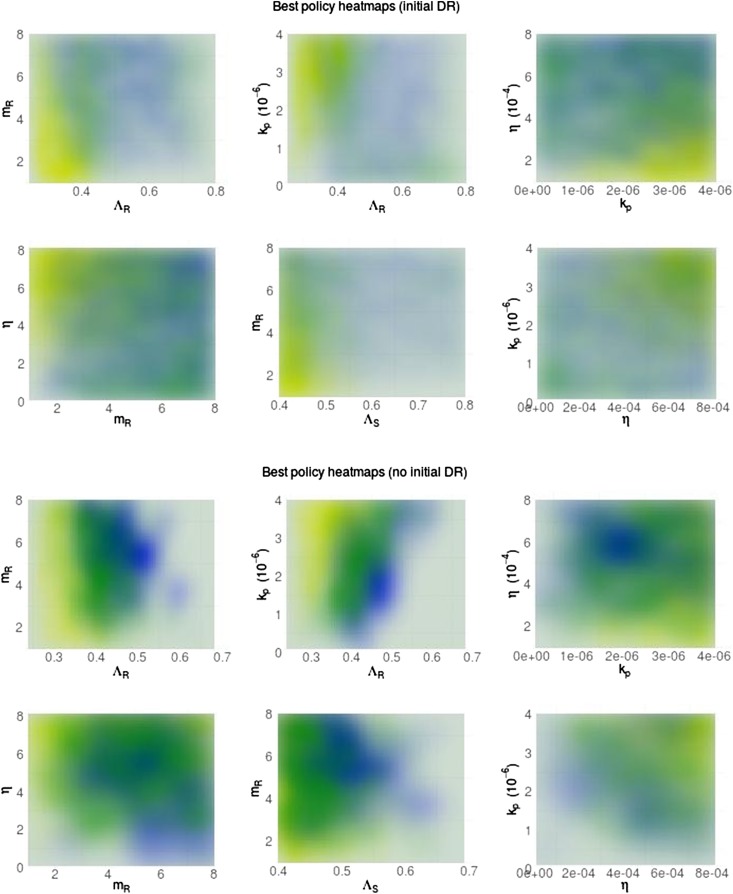
Heatmaps illustrating whether aggressive therapy (yellow), moderate therapy (blue) or neither, conclusively (neutral; green) minimize resistance. (Top set of panels) Pre-existing resistance means that the ‘moderate is best’ conclusion is spread out more widely over the parameter space; the DR strain does not benefit as much from DS growth. (Bottom set of panels) Without pre-existing resistance, there is more clear separation between the parameter regimes. Overall, the results are the same as in the main text: a moderate outcome is driven by high effective competition between strains, meaning a higher Λ_*R*_, *m*_*R*_ and lower values of immune parameters *k*_*p*_ and *η* (eta). **DOI:** http://dx.doi.org/10.7554/eLife.10559.011

**Appendix figure 2.**
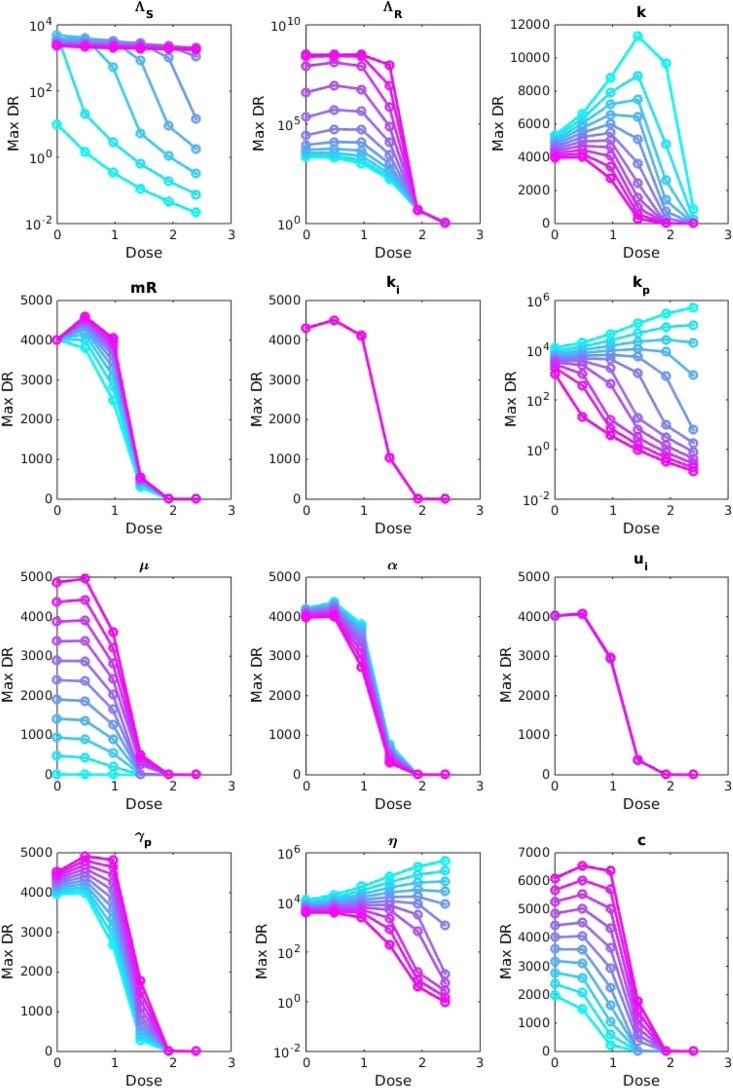
Sensitivity analysis, varying one parameter at a time. For each value of each parameter, the dosage was increased from 0 to 2.5 μg/ml per day, and the maximum number of DR bacilli during the infection was recorded. The baseline values of the parameters (when they were not varied) are: Λ_*s*_ = 0.5, Λ_*r*_ = 0.45, *m*_*R*_ = 2.8 and the remainder are as in Table 1. **DOI:** http://dx.doi.org/10.7554/eLife.10559.012

**Appendix figure 3.**
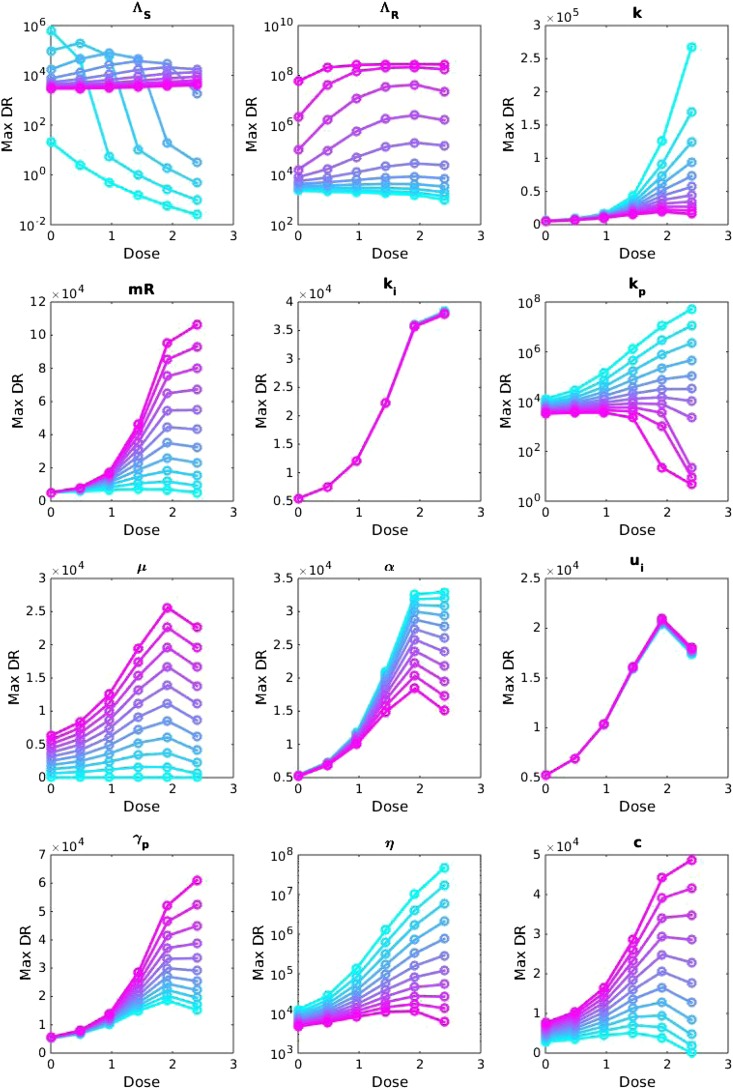
Sensitivity analysis, varying one parameter at a time. For each value of each parameter, the dosage was increased from LOW to HIGH, and the maximum number of DR bacilli during the infection was recorded. The baseline values of the parameters (when they were not varied) are: Λ_*s*_ = 0.6, Λ_*r*_ = 0.53, *m*_*R*_ = 3.4. Other parameters are *k* = 48, *k*_*p*_ = 2.2*e*^−6^, *k*_*i*_ = 7.2*e*^−4^, *μ* = 4*e*^−7^, *α* = 0.018, *σ*_*i*_ = 1000, *σ*_*p*_ = 10000, *γ*_*p*_ = 0.001 and the rest as in Table 1. The approach is the same as that in Appendix figure 2, except that the baseline set of parameters is different. **DOI:** http://dx.doi.org/10.7554/eLife.10559.013

If resistant cells are present initially, then they do not need to emerge by (rare) mutation or acquisition mechanisms from the sensitive strain. This simple observation has consequences for our analysis; a ‘moderate is best’, or neutral conclusion is much more likely with pre-existing resistance, keeping everything else the same. When there is no pre-existing resistance, the DR strain must have a higher MIC, higher growth rate and face a weaker immune system in order to be a robust competitor than it does when it is present initially. Appendix figure 1 shows the heatmaps as in Figure 4 but stratified according to whether there is pre-existing resistance.

To understand which policy minimizes resistance, one must be able to characterize the *net* effect that the presence of one strain has on the other strain. There is strong opportunity for competition between strains encoded in the model; competition plays out through shared resources which may be limiting as well as through the triggering of an immune response that suppresses both strains equally. Both of these effects occur when bacterial populations are large. However, the initial appearance of resistance also depends critically on the presence of drug-sensitive organisms. Altering both the strength of competition and the dependence of the DR strain on the DS progenitor population determines whether or not such competition is *effective*. In particular, effective competition naturally requires a DR strain that has the capacity to be a robust competitor to the DS progenitor. This can be achieved in two ways: it can maintain a strong growth capacity in the presence of antibiotic treatment or immune pressure, or it can face an immune system that is not particularly strong. Our exploration of the parameter space uncovered both of these mechanisms. These findings are not an artifact of the model structure, and indeed they will likely occur in any model that includes de novo appearance of resistant strains by mutation or acquisition of resistance determinants by drug-sensitive organisms, and where resistant strains can then compete for resources with their drug-sensitive cousins.

Effective competition can be described as the net extent to which a decrease in one strain benefits the other. This should capture both direct and indirect effects. Quantifying effective competition is challenging because either strain may affect the other over short or long time frames and because different ways to decrease one strain have different effects. From the second equation of (1), the direct effect of a decrease in *B*_*s*_ on the immediate growth of the resistant strain is simply the change in the acquisition term (*μB*_*s*_). If the other terms are small, this term can make the difference between net growth and net decline of resistant cells. The indirect effects are more difficult to determine; a decrease in *B*_*s*_ will mean more available resource and reduced recruitment of immune cells which will have onward effects. We made a step towards quantifying effective competition as follows. We numerically solved the system, obtaining values of each variable through time. We decreased *B*_*s*_ and computed the predicted value of *B*_*r*_ two time steps later, using Euler's method, the derivatives in (1) and the time steps defined by the adaptive ODE solver. We did this (separately) at each time point and averaged the fractional change in *B*_*r*_ due to a 5% change in *B*_*s*_ over the portion of the trajectory where *B*_*s*_ > 10^5^. We do this only in the untreated case, for each value of the parameters we sampled. This captures only the immediate indirect effects (occurring in the next two small time steps) and the intervention we made (reducing *B*_*s*_ when dosage is 0) is not the same as treatment. Nevertheless, this formulation of effective competition is a good predictor of whether treatment, when added, increases resistance. We used it to classify whether treatment would increase or decrease resistance, and found that the classification worked well, with an area under the receiver–operator characteristic curve (AUC) of 0.94 (the theoretical maximum is 1, and a classifier that guesses randomly has AUC of 0.5). See Appendix figure 10.

### Between-host model

**Appendix figure 4.**
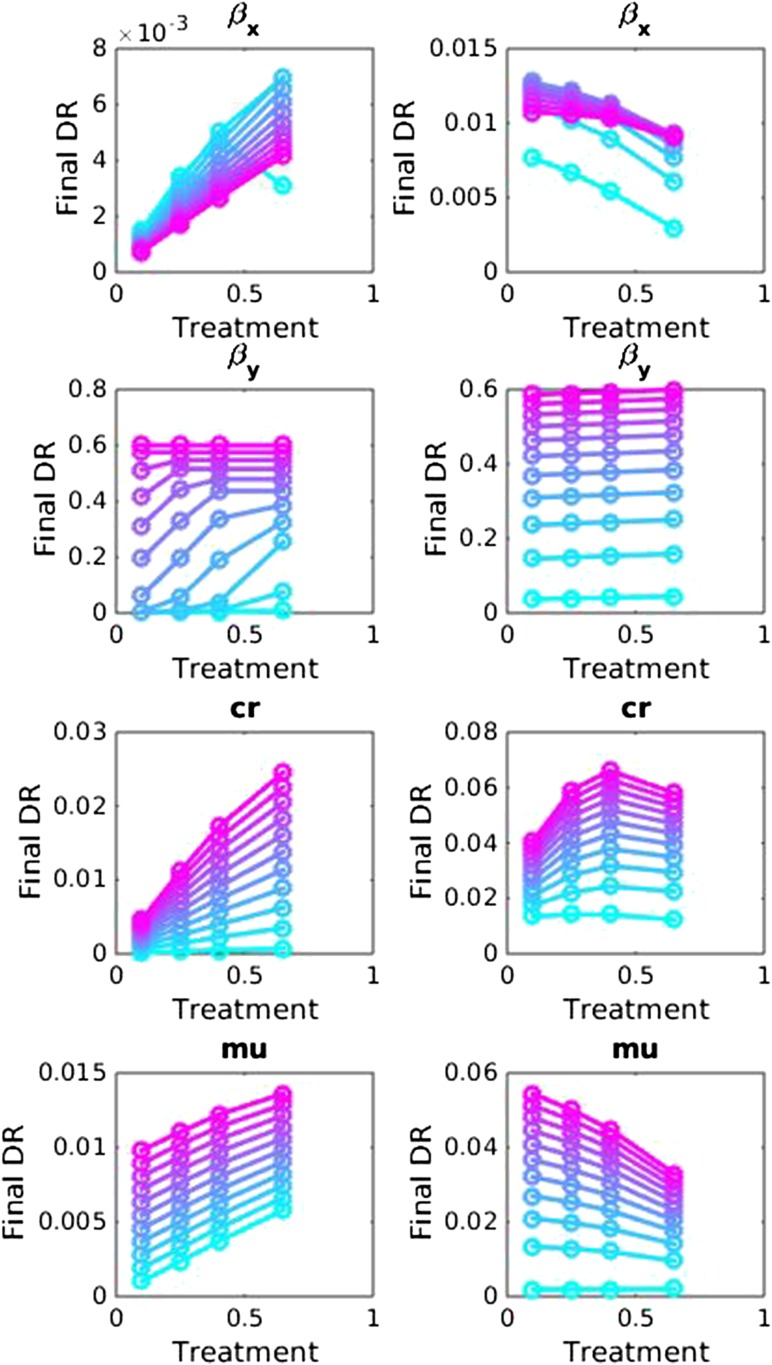
Sensitivity analysis, varying one parameter at a time in the between-host model over the ranges listed in Table 1. Left side: baseline parameter values are *β*_*x*_ = 1.5, *β*_*y*_ = 1.04, *c* = 0.05, *r* = 0, *μ* = 0.001. Right side: baseline values are *β*_*x*_ = 2, *β*_*y*_ = 1.1, *c* = 0.3, *r* = 0.05, *μ* = 0.0001. **DOI:** http://dx.doi.org/10.7554/eLife.10559.014

**Appendix figure 5.**
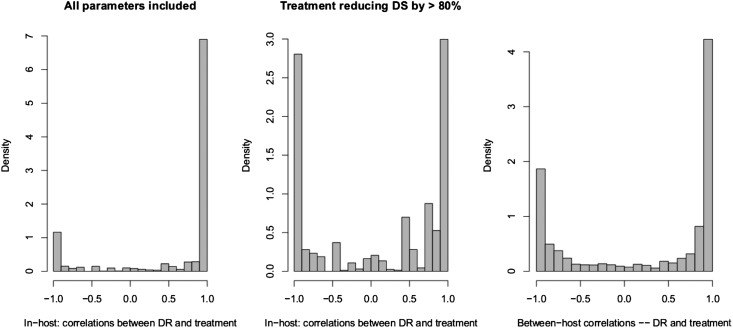
Spearman correlations between treatment level and maximum resistance in both models. (Left) In-host model. Left panel shows all parameters. Right panel shows only those for which treatment succeeds. The difference is that where treatment does not succeed, it drives resistance. (Right) Population-level model. **DOI:** http://dx.doi.org/10.7554/eLife.10559.015

Figure 5 illustrates the behavior of the between-host model at two demonstrative parameter sets, one illustrating resistance decreasing with treatment due to the dominant effect of acquisition of DR from the DS strain, and the other illustrating resistance increasing with treatment as a result of inter-strain competition. Appendix figure 4 shows the relationship between resistance and strength of treatment and its sensitivity to variation in single parameters. No one parameter defines whether treatment increases or decreases resistance.

The model has the possibility of three distinct steady states (equilibria): no disease, both DS and DR present, and only the DR strain present. If there were no acquisition of resistance, there would also be the possibility of an equilibrium with only the DS strain. Figure 5 shows that the stability region of the DR-only equilibrium changes as the similarity coefficient increases. When *c* = 0, the DR-only equilibrium is invaded by the DS strain as soon as *R*_01_ > 1 (vertical blue line). However, when the strains are more similar, *R*_01_ must be higher in order to invade, and when *c* = 1, there is no equilibrium DS prevalence unless *R*_01_ ≥ *R*_02_.

**Appendix figure 6.**
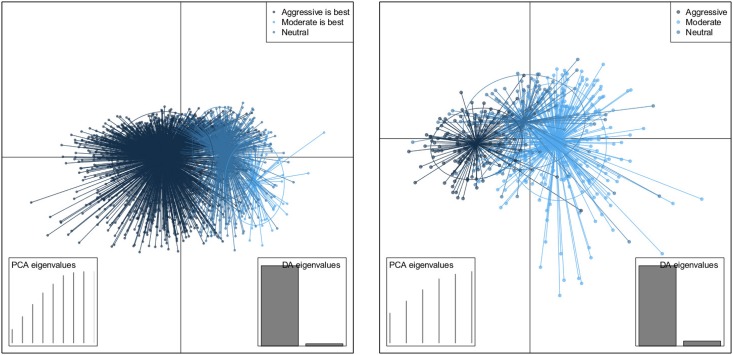
DAPC scatterplot for the within-host model. The x-axis is a linear combination of parameters corresponding to the primary DAPC discriminant function, and the y-axis corresponds to the second. Accordingly, the groups are plotted by their positions on the first two DAPC axes. The relative portions of the variance that these axes capture is illustrated in the DACP eigenvalue plot (bottom right corner). Almost all of the difference between the groups is captured by the x-axis (high gray bar in the bottom left plot, compared to a low gray bar for the variation captured by the second axis). The PCA eigenvalues plot in the bottom right shows the portion of variance retained when keeping *k* PCA axes. In both models, the neutral group, in which treatment was not strongly correlated with the level of resistance in either direction, lies directly between the aggressive and moderate groups. **DOI:** http://dx.doi.org/10.7554/eLife.10559.016

**Appendix figure 7.**
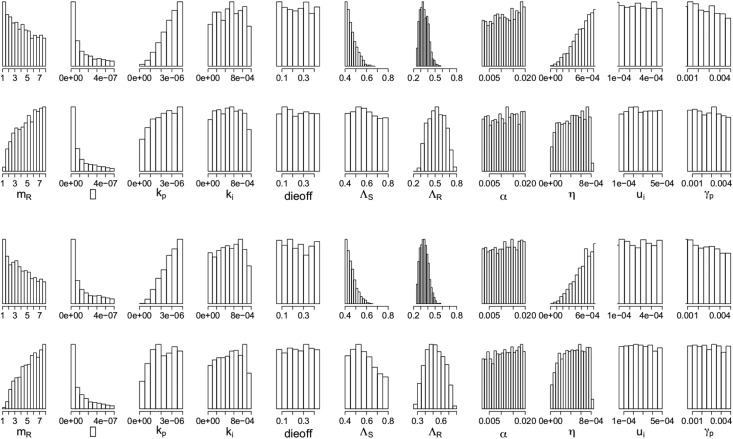
Parameters from the two regimes: (a) with the lysis term and (b) without it. In each set, the top row shows the parameters under which resistance decreases with increasing dosage, and the bottom row shows those for which resistance increases with increasing dosage. The relationships between the two outcomes (treatment increasing or decreasing resistance) are unaffected by whether the lysis term is included. Removing the term is akin to including lysis in the equation for the resource usage, but with an *e* coefficient of 0; a more general model could include this with a coefficient *e*_*l*_ < *e* which would model the fact that resource consumption and release could happen with different efficiencies. Though we do not know what an appropriate choice for *e*_*l*_ would be, the results with *e*_*l*_ = 0 (bottom set of histograms) are the same as those with *e*_*l*_ = *e* (original model; top set of histograms). **DOI:** http://dx.doi.org/10.7554/eLife.10559.017

**Appendix figure 8.**
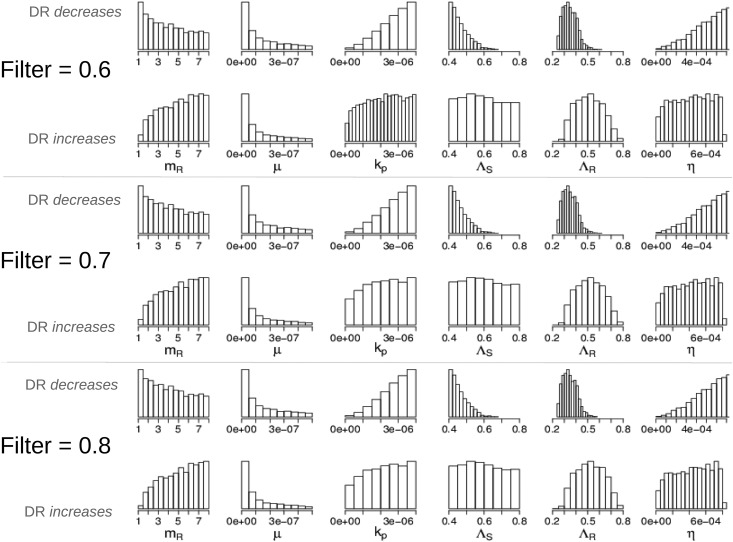
Parameters in the two modes showing that the filter defining ‘effective treatment’ does not change the relationships between parameters and whether treatment increases or decreases resistance. We have shown only the parameters whose distributions are different in the two modes (compare to Appendix figure 7). **DOI:** http://dx.doi.org/10.7554/eLife.10559.018

**Appendix figure 9.**
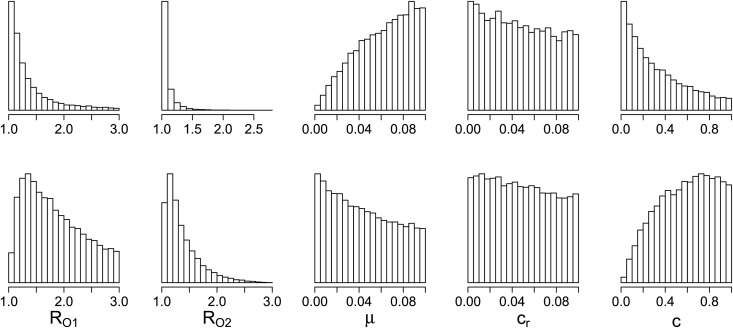
Parameters where the two contrasting policies work best in the population level model. Top row: treatment decreases resistance. Bottom row: treatment increases resistance. **DOI:** http://dx.doi.org/10.7554/eLife.10559.019

Appendix figure 9, Figures 6, 7 illustrate how the best policy depends on the fitness of the DR strain and the other parameters. Treatment decreases resistance when the *R*0 values of both strains are relatively low, the rate of acquisition of resistance is high and the similarity coefficient is low. We find that the parameter groups where aggressive therapy minimizes resistance are well separated by those where moderate therapy is best, by a single DAPC function (Appendix figure 6). Here, the strongest driver of a moderate policy being best is a high similarity coefficient (*c*, coefficient 0.93). High *R*_02_ and *R*_01_ (coefficients 1.01, 0.65) contribute, as does a low acquisition rate (coefficient −0.56). Somewhat surprisingly, the rate of competitive release does not contribute to the DAPC weighting (−0.02).

An aggressive strategy is more likely to minimize resistance when the DR strain is relatively unfit (low *R*_02_), and the DR population is supported by a high rate of acquisition. Furthermore, an aggressive strategy is likely to be best when the strains are more independent (a low similarity coefficient). Independence means that even a relatively unfit strain can be transmitted in the population, despite the presence of a more fit strain, because when strains are independent they can each super-infect hosts already infected with the other strain, and they can be transmitted from those with dual infection (if these cannot happen then the strains cannot be independent; rather, they would compete for hosts and/or for infectivity). We noted previously (Colijn et al., 2009) that such co-infection can, but does not always, allow DR strains to persist in the long term where they would not be able to do so otherwise; similar results were recently reported by Hansen and Day (2014). Our current results clarify that these effects are a result of the level of competition and are not a consequence of co-infection. Co-infection can be present under high, low, or intermediate levels of competition.

The factors that favor an aggressive policy—lower *R*_02_, higher rates of resistance acquisition and increased independence between strains—have the net impact of reducing inter-strain competition. A low *R*_01_ also makes an aggressive approach more likely to be preferred (see Appendix figure 9); competition for hosts is low when there are plenty of susceptible hosts, whether the model has strict competition mechanisms or not. This occurs when both *R*_0_ values are low. Low *R*_02_ means the DR strain is not a fit competitor, a high independence (1 − *c*) explicitly reduces competition through protection from re-infection and through independent recovery, and a higher mutation rate increases the benefit the DR strain enjoys from DS.

**Appendix figure 10.**
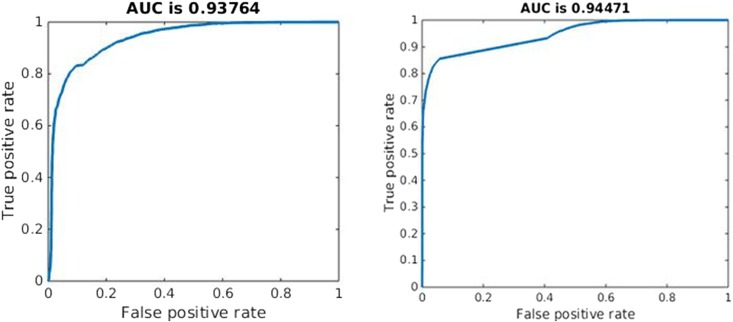
Quality of classification using the instantaneous measure of effective competition to classify a parameter set according to whether treatment will increase or decrease resistance. A random classifier has an AUC of 0.5. We used the sign of the Spearman correlation between treatment and resistance as the ‘true’ classification (competing if treatment increases resistance), and the value of our effective competition quantity as the ‘scores’. Curves were created using the perfcurve function in matlab. **DOI:** http://dx.doi.org/10.7554/eLife.10559.020

We used the same approach to quantify the short-term competitive interaction as in the within-host model. We reduced the population of the sensitive strain, iterated the model forward two time steps, and computed the effect on the resistant strain (in the absence of treatment). We used the average proportional change in the resistance strain following this direct reduction in the sensitive strain to predict what would happen (increase or decrease) to resistance under treatment. As in the within-host model, this measure of effective competition is a good predictor of the effect of treatment (AUC 0.94; Appendix figure 10).

The models are directly linked through the competitive release, *r*, which reflects a portion (in the between-host model) of treated sensitive infections that convert to resistant ones. Even where this does not occur at all (*r* = 0), successful treatment of individuals drives population-level resistance when strains are competing. Consider the within-host model in the regime where aggressive treatment minimizes resistance, for example, low DR growth Λ_*r*_ or a strong immune system, or high rate of acquisition of resistance. Individuals treated successfully are not susceptible to, or infectious with, the sensitive strain. At the between-host level, though, they may remain susceptible to a circulating resistant strain, which now has more susceptible hosts available than the sensitive strain. Treatment (reducing *R*_01_) moves the system to the left on the invasion plot (Figure 5C), increasing resistance if there is effective competition at the between-host level.

## Discussion

An aggressive policy for antibiotic treatment is preferred when the appearance and persistence of DR is driven by the existence of a sufficiently large DS population. In these settings, the benefits to the DR population which accrue from the acquisition of resistance from DS outweigh the costs of competition from a larger DS population. In contrast, a moderate dosing policy is preferred when the DR strain is a fit enough competitor that acquisition of resistance plays a sufficiently small role in the DR population dynamics. Here, the cost of competition from the DS population outweighs the benefit of additional DR bacteria appearing through acquired resistance. Both modes occur in models containing acquisition of resistance and competition, and a small change in parameters can shift models from one mode to the other. Understanding why previous models and theory have differed in support of aggressive (Ehrlich, 1913; Knudsen et al., 2003; Wiuff et al., 2003; Tam et al., 2005; D'Agata et al., 2007; Ankomah and Levin, 2014; Kim et al., 2014) and moderate (de Roode et al., 2004; Wargo et al., 2007; Gullberg et al., 2011; Huijben et al., 2013) approaches requires evaluating both structural assumptions and parameter choices (Spicknall et al., 2013) as these together affect the strength of effective competition between DR and DS strains. We have used a comprehensive approach to exploring these assumptions and have begun the process of defining measures of *effective competition* to predict whether moderate or aggressive treatment minimizes resistance.

Previous contradictory results on this question fit neatly into the framework we have presented. In Ankomah and Levin (2014), the model structure incorporates complex interactions between strains, allowing for many facets of competition to be explored. At their chosen parameter values, however, there is little effective competition between DR and DS strains and they found an aggressive policy to be best. In a recent work by Kim et al. (2014), DR strains had two alleles with no onward fitness evolution, little in-host competition, and low DR fitness; consequently, they also found that an aggressive approach would be best. Another recent model (Gomes et al., 2013) assumed that there was competition for resources at high bacterial populations, and concluded that this competition could play a role in suppression of resistant strains. Work by Geli et al. (2012) explored different ecological dynamics and found that strong immunity supports an aggressive policy, but that selection was most intensive at intermediate strengths of treatment in chronic infections (Geli et al., 2012; Kouyos et al., 2014). Gullberg et al. (2011) found that even low concentrations of antibiotic (where the DR and DS fitness may not differ) can rapidly enrich DR sub-populations. Huijben et al. (2013) found experimentally that competitive release of (pre-existing and relatively fit) resistant strains increased with increasing drug pressure.

We conclude that both of these perspectives are reasonable. We find that even in a system where aggressive approaches are most frequently best (the within-host model, based on Ankomah and Levin (2014)), a moderate approach can be preferred if the DR strain is slightly more fit and the environment is slightly more permissive; in this case, inter-strain competition mechanisms, which are always present, are more effective. Likewise, at the between-host level where we might expect herd-level competition effects to play out, we find that even where competition mechanisms are strong, an aggressive approach may be best of one or both strains have a low basic reproduction number or if the rate of acquisition of resistance is high. Both of these factors limit the effective competition.

In most circumstances, we expect that DR and DS variants of a single pathogen compete quite strongly: they will be closely related, and so are likely to share antigenic properties and induce a similar host immune response. They are also likely to consume or be reliant on similar host resources (Fiegna and Velicer, 2005) and occupy similar biological niches within hosts (Dall'Antonia et al., 2005; Lam and Monack, 2014). The extent to which these aspects dominate the fact that resistance is also driven by de novo acquisition, and hence benefits from high DS population levels, will depend on the acquisition rate and mechanism (Lipsitch and Samore, 2002) as well as on the degree of effective competition. Furthermore, models have typically reflected competition for host resources or via a carrying capacity (Geli et al., 2012; Gomes et al., 2013; Ankomah and Levin, 2014), such that competition takes place at high bacterial populations. If direct antagonistic (Claverys and Håvarstein, 2007; Baquero and Lemonnier, 2009) or cooperative (Diard et al., 2013) interactions occur, they are likely to substantially alter the extent and timing of competition, with profound onward consequences for optimal treatment.

We do want to emphasize some key differences between the problem of minimizing resistance among each individual receiving treatment and minimizing resistance of circulating pathogens in a community (Lipsitch et al., 2000; Lipsitch and Samore, 2002; Cohen et al., 2006; zur Wiesch et al., 2011; Geli et al., 2012; Mills et al., 2013). Consider, for example, an immune-competent individual initially harboring a drug-susceptible infection free of any (or many) sporadically resistant isolates. An aggressive approach may well be preferred for this individual. However, if there is a fit resistant strain circulating in the community, then this policy can drive substantial resistance at the population level (e.g., by selectively suppressing the DS strain, making hosts susceptible to re-infections only with the DR strain), even if it does not increase the risk of acquiring resistance in any *individual* given treatment. Consequently, an aggressive approach might well be best at the individual level while still driving resistance over longer time frames. An aggressive approach may also diminish in utility over time if DR strains become fitter through selection, if they begin to circulate widely and compete with DS strains (zur Wiesch et al., 2011).

Co-infection of individual hosts by multiple strains or isolates has been observed for most pathogens in which it has been investigated (Balmer and Tanner, 2011), and we have incorporated it in both models. At the population level, models that fail to include co-infection assume that co-infections do not occur; this equates to a very strong assumption about competition for hosts, regardless of whether there is sufficient data to inform co-infection parameters.

We have previously argued that diversity-promoting mechanisms in models should be explicit (Lipsitch et al., 2009) and that ‘neutral’ models are a useful framework for understanding implicit assumptions in multi-strain models. Here, we note that such neutrality *is* competition. The ‘no coexistence for free’ directive can be reframed: we expect identical strains to compete. The extent of competition is a key driver of how the balance between multiple strains changes in response to interventions; if we are to use models to understand these responses, we must be clear about the mechanisms and the extent of effective competition between strains.

These results highlight the importance of identifying empirical data that reveal whether effective competition between DS and DR strains is present. Experimental approaches in which mixed bacterial populations are studied in vitro or in vivo may reveal mechanisms by which these sub-populations may exhibit interference competition through direct interaction (Claverys and Håvarstein, 2007) or exploitative competition through shared dependence on a common resource (Fiegna and Velicer, 2005). These types of controlled experiments have been valuable for identifying conditions under which such direct competition effects are likely to manifest within individual hosts. Identifying data that would reveal the conditions under which we would expect competition between DS and DR strains at the community level is clearly more challenging. The scale and the timing at which we would expect to observe the effects of intraspecific competition will likely differ by pathogen type. Similar to studies of vaccines or other interventions in which indirect effects are important to consider, community-randomized trials are the most promising design, but the expense and logistics of such trials for considering different antibiotic dosing strategies may be prohibitive.

In the absence of such trials, relating population-level antibiotic use data to surveillance data describing trends in resistance in the community may help to identify signals of such competition. Detailed analysis of the numbers and ages of treated cases, the population density, ‘drug-bug’ interactions, and the time since resistance first emerged (Turnidge and Christiansen, 2005) could improve our ability to do this. Meanwhile, careful consideration of the level of effective competition is essential when using models to understand the relationship between antimicrobial use and resistance.

## Appendix 1

Between-host model: two ends of a continuum Here, we compare a model with two very different, independent pathogens circulating in a community, and the 2-strain neutral null model (Lipsitch et al., 2009). Previously (Lipsitch et al., 2009; Colijn et al., 2010), we argued that neutrality is required in order for a model of multiple strains to make biological sense when the strains are identical: in this case, it should not matter which strain an individual or sub-group is infected with. The dynamics of the total prevalence should depend only on the numbers of hosts infected. Furthermore, each strain should have the same ability to cause new infections and should not be comparatively advantaged or disadvantaged by being rare. Otherwise, an arbitrary re-labeling of some cases (all of whom have identical strains by assumption) alters which of these cases seed new infections. These two conditions constrain the equations of any multi-strain model. First, if dual infection can result from re-infection, then re-infection must also be able to out-compete one of the strains in a dually infected host, leading back to single infection. Otherwise, dual infection can protect a rare strain (Lipsitch et al., 2009; Colijn et al., 2010), as was also noted recently in Hansen and Day (2014). Second, in the limit in which the infections are identical, recovery from one and not the other is not logical; dually infected hosts recover back to susceptible. Finally, dually infected hosts contribute the same *total* infectiousness to the force of infection, divided equally between the strains (This assumption can be relaxed while preserving neutrality, but doing so requires also modeling hosts with multiple single infections [Lipsitch et al., 2009].). We found (Lipsitch et al., 2009; Colijn et al., 2010) that models that reduce to this neutral interaction when strains are identical also do not promote coexistence of diverse strains. Here, we have developed a model that incorporates this neutral null limit when the strains are very similar, but which also allows the possibility that strains are very different, to the point of not interacting with each other at all. Different strains might inhabit very different niches within hosts, trigger different immune responses, act over different time periods, be transmitted through different routes, and so on. When strains are independent, new infection events do not displace existing strains, but add to them. Hosts clear strains independently, so that when a dually infected host recovers from one strain, they remain (singly) infected with the other. Finally, dually infected hosts are equally infectious with each strain as they would be if they didn't have the other strain. These requirements also constrain the equations of a model representing the two strains. We have developed a model that captures these very different limiting interactions and allows us to explore the continuum between them. A (SIS, or susceptible-infected-susceptible) model with two entirely independent strains must look something like this:

(3) $$\begin{array}{l} \frac{dS}{dt}=B-F_{x}S-F_{y}S-uS+u_{x}X+u_{y}Y\\ \frac{dX}{dt}=F_{x}S-F_{y}X+u_{y}D-u_{x}X\\ \frac{dY}{dt}=F_{y}S-F_{x}Y+u_{x}D-u_{y}Y\\ \frac{dD}{dt}=F_{y}X+F_{x}Y-u_{x}D-u_{y}D, \end{array}$$

with *F*_*x*_ = *β*_*x*_(*X* + *D*), *F*_*y*_ = *β*_*y*_(*Y* + *D*) and *B* a birth term. For independence, we require that the total prevalence of each strain evolves as it would without the other strain. For example, the total prevalence of *X* is *T*_*x*_ = *X* + *D* and the number susceptible to strain *X* is *S*_*x*_ = *Y* + *S* = 1 − *T*_*x*_. Adding the second and fourth equations of Equation 3, we have

$$\frac{d}{dt}\left(X+D\right)=\beta_{x}\left(X+D\right)\left(S+Y\right)-u_{x}\left(X+D\right),$$

which is

$$\frac{dT_{x}}{dt}=\beta_{x}T_{x}S_{x}-u_{x}T_{x},$$

namely exactly the single-strain SIS model. We have the analogous expression for *Y*. Solving these de-coupled equations for *T*_*x*_ and *T*_*y*_ is equivalent to solving Equation 3, so Equation 3 describes independent dynamics. Furthermore, Equation 2 reduces to Equation 3 when the similarity coefficient *c* is 0 (and *T* = 0). When the strains are very similar (*c* = 1), Equation 2 in the main text reduces to Equation 14 of Lipsitch et al. (2009) with (their) *c* = *q* = 1/2. That model was shown to be neutral through its equivalence with Equation 1 in the same work. This is the origin of the terms in Equation 2 with $\frac{1}{2}c$ in them; when our similarity coefficient is 1, these terms become the relevant terms in Lipsitch's model (14) for (their) *c* = 1/2. The models differ in their re-infection from the dual class (0 where the similarity coefficient is 0) and in the clearance terms from the dually infected hosts. To obtain those clearance terms in Equation 2, we interpolate: We have three potential clearance terms: (1) dual to *X*, (2) dual to *Y*, and (3) dual to *S* (not shown in Equation 2 because *S* = 1 − *X* − *Y* − *D*). When *c* = 0, these terms need to be *u*_*x*_*D*, *u*_*y*_*D*, and 0, respectively. When *c* = 1, they need to be 0, 0 and $\frac{1}{2}\left(u_{x}+u_{y}\right)D$, where the latter means that when the strains are identical the clearance is correct. Interpolating (with terms (1), (2), (3) in vector notation), we have these three terms as $\left(1-c\right)\times \left(u_{x}D,u_{y}D,0\right)+c\times \left(0,0,\frac{1}{2}\left(u_{x}+u_{y}\right)D\right)$. This yields the model in Equation 2. We note that the result of this interpolation is related to recent work on coinfection (Hansen and Day, 2014), in which the authors ask how co-infection may drive increase in resistance (through an increased ability *R*_*r*_ of the DR strain to invade a DS-strain's equilibrium, in the dynamical sense). They find that co-infection can increase *R*_*r*_ more when the DR strain is more productive within co-infections (has a higher contribution to the force of infection), and when it has a higher ability to co-infect the DS strain. Our results are consistent with this, but our framing points out that these effects occur because of how these aspects of co-infection modify the net competition between the strains. We verified numerically that the model has independent SIS dynamics for each strain when *c* = 0. We also verified that when *c* = 1, *T* = 0 and the parameters for the two strains are identical, the model is neutral in the sense of Lipsitch et al. (2009). When the parameters are not identical but *c* = 1, the model exhibits competitive exclusion. When *c* = 0, the two infections do not interact with each other—each spreads as it would without the other one present. A hypothetical example might be pneumococcal carriage and human papillomavirus (HPV). They likely do not have much immune response in common, and both can be asymptomatic and would likely not affect each other's transmission. They have very low similarity. This is at one end of a spectrum, and modellers do not choose to model transmission of these infections simultaneously (precisely because they would not affect each other). However, low values of *c* are implicitly assumed by models (of the same infection with different strains, e.g., resistant and sensitive) that do not take competition into account, for example, by including co-infection in a way that dramatically reduces competition (see also the discussion in Nicoli et al. (2015)). The final model in (2) contains, in the first equation of (2), terms for: entry from susceptibles into *X* (*F*_*x*_*S*), re-infection of *X* with the *Y* strain (*κF*_*y*_*X*), re-entry to *X* from duals re-infected with $X\left(\frac{1}{2}\kappa_{t}cF_{x}D\right)$, clearance of *X* (*u*_*x*_*X*), clearance of the *Y* strain in duals (1 − *c*)*u*_*y*_*D*, and acquisition of resistance plus competitive release of resistance due to treatment (*μ* + *Tr*)*X*. The second equation is analagous and the third, for duals, describes their infection, re-infection back to singly infected states and clearance. Implementation Models were implemented in Matlab and solved with ode15s, which is a built-in ODE solver in Matlab capable of handling stiff systems. Stiffness was an issue in the within-host model because of the wide range of orders of magnitude of both the variables (with cell numbers reaching 10^10^) and the parameters, together with the sharp threshold on the DR growth at *B*_*r*_ = 30. This was used to model the possibility of stochastic die-off of low populations of DR, and incidentally also prevents growth from only a fraction of a cell, which would otherwise be possible when using a continuous model to approximate a discrete variable. Parameters were generated uniformly over the ranges shown, except for *μ* in the within-host model. There, log10(*μ*) was uniformly distributed, to explore lower mutation rates more deeply than higher ones. The parameter ranges for the within-host model all surround the values used in Ankomah and Levin (2014). Stratified bar plots and color density plots were created in ggplot2 in R. The smoothing and density estimation analysis for Figures 4, 7 and Appendix figure 1 was done with the stat_density2d function in ggplot2. Sensitivity analysis We performed single-parameter sensitivity analyses in both models. These demonstrate that most parameters, varied on their own over the permissive ranges we have used in the overall parameter exploration, do not typically move the system from the mode in which treatment decreases resistance to the mode where resistance increases (competition). Appendix figures 2, 3 illustrate single-parameter variation in the in-host model, and Appendix figure 4 illustrates it in the between-host model. Appendix figure 5 illustrates that in the majority of cases there is a strong correlation between treatment strength and resistance, whether negative or positive. How parameter combinations affect the treatment–resistance relationship In the in-host model, whether a DR strain needs to emerge only after treatment begins, during a rise in bacterial burden in the DS strain, affects the inter-strain dynamics, because pre-existing resistance reduces the positive impact of the DS strain on the DR strain. We repeated the analysis underlying Figure 4, using only the subset with pre-existing resistance, and then only those results without pre-existing resistance. Other parameters ranged over the same intervals. Discriminant analysis of principle components (Jombart et al., 2010) (DAPC) finds combinations of variables that best separate groups (here, whether treatment increases or decreases resistance, or ‘neutral’). DAPC results indicated that almost all of the difference between the groups can be accounted for using a single discriminant function, both in the within-host model and the between-host one. Appendix figure 6 shows the groups plotted on the first two discriminant axes, illustrating the separation of the groups. The horizontal axis is the first discriminant function whose higher coefficients are given in the main text. Appendix figures 7, 8 show the parameters of the in-host model, selected according to whether an aggressive or a moderate treatment approach is best to minimize resistance. In the top rows, the aggressive policy is preferred (treatment decreases resistance); we see a lower MIC of the DR strain (labeled mR), a stronger immune system (higher *k*_*p*_ and *η* (eta)), a lower Λ_*r*_ and also lower Λ_*s*_. The lower Λ_*s*_ results in fewer DS bacilli, so fewer DR bacilli are created through mutation, allowing an aggressive policy to prevent their emergence. Appendix figure 9 shows the histograms and scatter plots for the between host model. These illustrate the same points made in the main text but again show the relationships between the parameters that were varied. An aggressive policy is most likely best when the DR strain has a relatively low basic reproductive number *R*_02_, and when the similarity coefficient is low. A low similarity coefficient means that competition is reduced, because the extent to which the strains are interacting is lower than when strains are very similar. Finally, Appendix figure 10 shows that the measure of effective competition defined in the main text can predict the relationship between treatment and resistance, in both the within- and between- host models.
